# Metal to insulator transition for conducting polymers in plasmonic nanogaps

**DOI:** 10.1038/s41377-023-01344-7

**Published:** 2024-01-01

**Authors:** Yuling Xiong, Rohit Chikkaraddy, Charlie Readman, Shu Hu, Kunli Xiong, Jialong Peng, Qianqi Lin, Jeremy J. Baumberg

**Affiliations:** 1 NanoPhotonics Centre, Cavendish Laboratory, Department of Physics, University of Cambridge, Cambridge, CB3 0HE UK; 2 School of Physics & Astronomy, University of Birmingham, Edgbaston, Birmingham, UK; 3 College of Advanced Interdisciplinary Studies and Hunan Provincial Key Laboratory of Novel Nano-Optoelectronic Information Materials and Devices, National University of Defense Technology, Changsha, China; 4 Hybrid Materials for Opto-Electronics Group, Department of Molecules and Materials, MESA+ Institute for Nanotechnology, Molecules Center and Center for Brain-Inspired Nano Systems, Faculty of Science and Technology, University of Twente, Enschede, Netherlands

**Keywords:** Nanocavities, Polymers, Nanophotonics and plasmonics

## Abstract

Conjugated polymers are promising material candidates for many future applications in flexible displays, organic circuits, and sensors. Their performance is strongly affected by their structural conformation including both electrical and optical anisotropy. Particularly for thin layers or close to crucial interfaces, there are few methods to track their organization and functional behaviors. Here we present a platform based on plasmonic nanogaps that can assess the chemical structure and orientation of conjugated polymers down to sub-10 nm thickness using light. We focus on a representative conjugated polymer, poly(3,4-ethylenedioxythiophene) (PEDOT), of varying thickness (2-20 nm) while it undergoes redox in situ. This allows dynamic switching of the plasmonic gap spacer through a metal-insulator transition. Both dark-field (DF) and surface-enhanced Raman scattering (SERS) spectra track the optical anisotropy and orientation of polymer chains close to a metallic interface. Moreover, we demonstrate how this influences both optical and redox switching for nanothick PEDOT devices.

## Graphical abstract

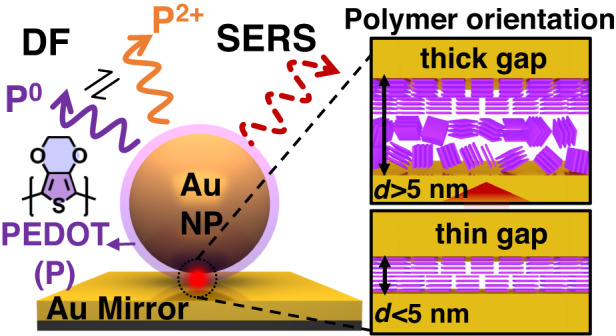

## Introduction

Conjugated polymers with delocalized electrons along their backbone possess conductivities and colors that are easily modified by doping and dedoping^1^. These tunable electrical and optical properties, combined with easy processability, mechanical flexibility, and a wide range of possible molecular functionalization, make them the major active components across a wide span of future technologies including flexible and emissive displays, organic (bio)electronics, and wearable sensors. The performance of conjugated polymers is closely related to their structural conformation, showing both electrical anisotropy^1^ with higher charge carrier mobility along the polymer backbone, and optical anisotropy^1–3^ for light polarizations oriented with respect to the polymer packing. The study and control of their microstructure is thus critical for the design of high-performance functional devices.

On micro- and nano-scales (>100 nm), diffraction-based techniques such as grazing incidence wide-angle X-ray scattering and selected-area electron diffraction are employed to examine polymer orientation^1,4,5^. However, these tools are complex, expensive, and often destructive, and only provide information about the crystalline phases of polymers. While optical ellipsometry can assess refractive anisotropies of conjugated polymer films^2,3,6,7^, it lacks chemical or structural insights. Vibrational spectroscopy methods such as Raman and FTIR have thus gained research interest due to their capability to determine molecular orientation in both crystalline and amorphous polymer phases^4,8^. However, in thin films below 100 nm, and especially at critical interfaces influencing electron transport with neighboring materials, Raman and FTIR techniques face challenges with poor signal-to-noise. Consequently, studies of molecular orientation in polymers primarily depend on X-ray and electron diffraction^9,10^. Developing a facile optical technique to characterize polymer conformations, particularly at interfaces, would thus be immensely beneficial.

**Fig. 1:**
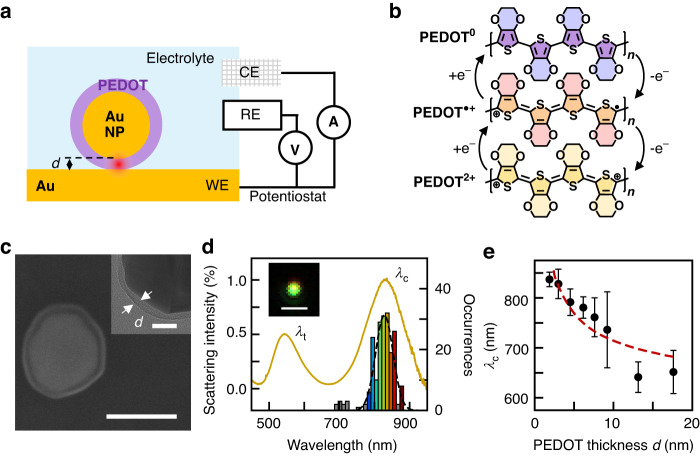
PEDOT in NPoM geometry. **a** Schematic of Au@PEDOT NPoM in spectro-electrochemical cell with three electrodes: gold mirror working electrode (WE), platinum counter electrode (CE) and Ag/AgCl (in 3 M KCl) reference electrode (RE). **b** Redox reactions of PEDOT: PEDOT^0^ = fully reduced; PEDOT^•+^ = half oxidized; PEDOT^2+^ = fully oxidized. **c** SEM image of Au@PEDOT. Scale bar: 100 nm. Inset: TEM image of PEDOT shell with thickness ${\boldsymbol{d}}$. Scale bar: 20 nm. **d** Dark-field scattering spectra for Au@PEDOT with ${\boldsymbol{d}}$ = 3 nm in air (${\boldsymbol{N}}$ = 200), with histogram of coupled plasmon mode ${{\boldsymbol{\lambda }}}_{{\bf{c}}}$ positions. Inset: scattering image of single Au@PEDOT NPoM. Scale bar: 1 μm. **e** NPoM coupled mode plasmon ${{\boldsymbol{\lambda }}}_{{\bf{c}}}$ *vs* PEDOT shell thickness ${\boldsymbol{d}}$, in air. Error bar from width of ${{\boldsymbol{\lambda }}}_{{\bf{c}}}$ histogram. All data for Au nanoparticle core of 100 nm diameter, dashed line is prediction from QNM model (Note S1)

Here we demonstrate a simple in-situ spectroscopic technique that provides both chemical and structural information for conjugated polymers in different redox states, giving sub-10 nm spatial data. This is achieved by confining light to volumes <100 nm^3^ within the polymer using a plasmonic nanogap geometry known as the nanoparticle-on-mirror (NPoM, Fig. 1a)^11^. Here, a widely used conjugated polymer PEDOT, whose backbone is representative of a class of valuable conjugated polymers^12,13^, is integrated into well-controlled 2-20 nm-thick nanocavities formed between Au nanoparticles and a Au mirror. The plasmonic resonance of this geometry is highly sensitive to the complex permittivity of the polymer in the gap^11,14,15^. This resonance also strongly enhances the optical field polarized perpendicular to the metal facets, accompanied by billion-fold increases in surface-enhanced Raman scattering (SERS) signals, thereby allowing direct chemical probing of ~1000 PEDOT monomer units. In combination with cyclic voltammetry (CV), this geometry shows fast-switching color dynamics^16^. Dark-field (DF) scattering spectroscopy tracks in real-time the electrochemical response of the conjugated polymer (Fig. 1b), while SERS uncovers the chemical structure and doping mechanism^17^. We find systematic changes in both DF and SERS spectra when reducing polymer thickness, which we attribute to changes of polymer orientation in these ultrathin layers.

## Results and discussion

### *e*NPoM optical switching with varying gaps

The electrochromic NPoMs (*e*NPoMs) are constructed by encapsulating 100 nm gold nanoparticles with a PEDOT polymer shell using surfactant-assisted in-situ chemical polymerization (Fig. 1c)^17^. By controlling the initial monomer concentration, Au@PEDOT core-shell nanoparticles with different shell thickness are obtained, as confirmed by dynamic light scattering (DLS) (Method, Fig. S1). The Au@PEDOT nanoparticles are deposited onto a gold mirror to form a plasmonic cavity, where the combined resonance of the gold nanoparticles and their image charges in the mirror strongly couple with each other, enabling extreme light confinement within the cavity ‘hotspot’.

The *e*NPoM nanocavities support various modes, including the transverse mode ${\lambda }_{{\rm{t}}}$ and coupled mode$\,{\lambda }_{{\rm{c}}}$, both measurable using DF scattering techniques (Fig. 1d). The transverse mode arises from a lateral dipole across the entire *e*NPoM, weakly confined and mainly influenced by the nanoparticle diameter^18–20^. In contrast, the coupled plasmonic resonance wavelength ${\lambda }_{{\rm{c}}}$ is sensitive to both the refractive index and the thickness $d$ of the gap material, as determined by the PEDOT shell, as well as the nanoparticle radius $R$. Typical DF spectra from a single *e*NPoM with 3 nm PEDOT shell (Fig. 1d) show a coupled mode wavelength ${\lambda }_{{\rm{c}}}\sim$ 835 nm, with its typical field distribution^20^ (Fig. S2) showing tight confinement within the gap. The histogram of the observed ${\lambda }_{{\rm{c}}}$ for 200 *e*NPoMs shows consistent coupled mode wavelengths with a normal frequency distribution that reflects the monodispersity of nanoparticle size (as found in DLS), and thus confirms the uniform *e*NPoM gap size across the sample.

The coupled mode wavelength consistently blue-shifts for increasing PEDOT shell thickness (Fig. 1e). This tuning trend aligns with the analytic estimates derived from a generalized circuit model for spherical NPoMs^11,21^,

1 $${\left({\lambda }_{{\rm{c}}}/{\lambda }_{{\rm{p}}}\right)}^{2}={\varepsilon }_{\infty }+2{\varepsilon }_{{\rm{d}}}+4{\varepsilon }_{{\rm{d}}}{\varepsilon }_{{\rm{g}}}^{\chi }\mathrm{ln}\left[1+\varsigma R/d\right]$$

where ${\lambda }_{{\rm{p}}}$ is the bulk plasma wavelength (for Au, ${\lambda }_{{\rm{p}}}\sim$ 148 nm), constants $\chi \sim 0.5,\varsigma \sim 0.1$, and ${\varepsilon }_{\infty },{\varepsilon }_{{\rm{d}}}$ and ${\varepsilon }_{{\rm{g}}}$ are the permittivities of Drude background for Au, the dielectric medium in which the system is embedded, and the material in the NPoM gap. Either a decrease in gap size (shell thickness) or increase in PEDOT refractive index induce redshifts in ${\lambda }_{{\rm{c}}}$ (Fig. 1e). We note that this model does not consider the nanoparticle facet size or shape^20^, both of which influence the optical coupled modes in the NPoM system and impact the prediction accuracy of the resonance wavelength. More accurate estimates (Note S1, Fig. S2b) confirm this trend (dashed line).

**Fig. 2:**
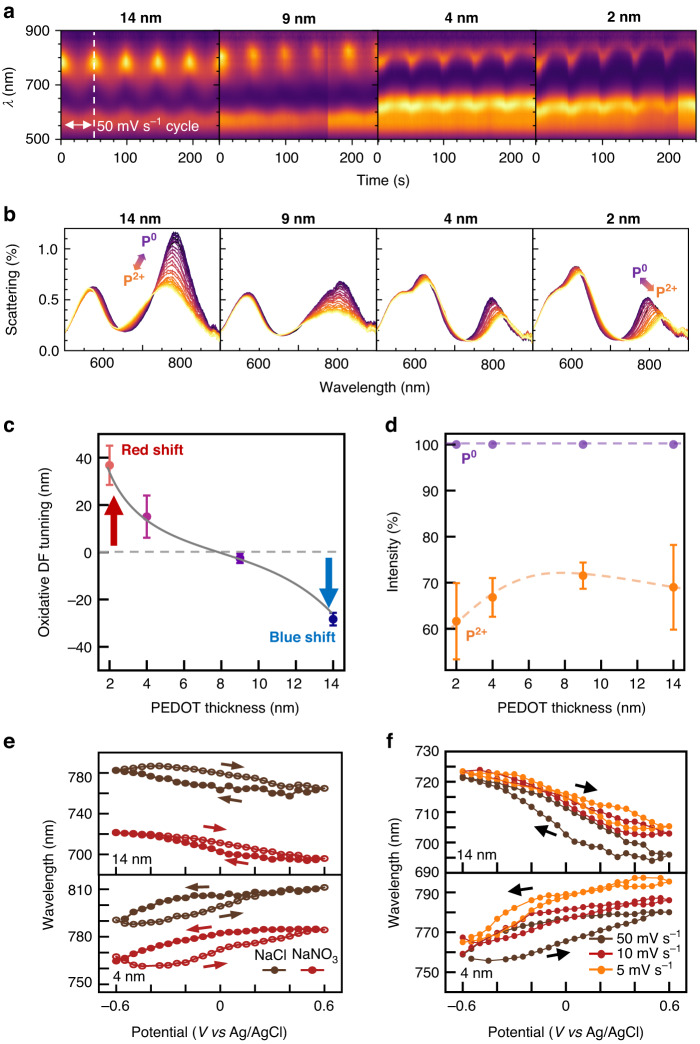
Electrochromic switching of Au@PEDOT eNPoMs. **a** Dynamic response in DF scattering of Au@PEDOT NPoMs with 2, 4, 9, 14 nm PEDOT shells in 0.5 M NaCl electrolyte. Scan rate: 50 mV s^−1^. **b** Corresponding DF scattering spectra of single NPoMs of each thickness. P^0^, PEDOT^0^; P^2+^, PEDOT^2+^. **c** Optical tuning and **d** corresponding intensity switching of NPoMs *vs* PEDOT thickness (gap size). **e** Tuning hysteresis and effect of different anions on DF scattering of NPoMs *vs* applied potential. Scan rate: 50 mV s^−1^. **f** Effect of scan rate on dynamic response in DF scattering of 4, 14 nm gap PEDOT NPoMs *vs* applied potential in 0.5 M NaNO_3_ electrolyte

Electrochemical redox of PEDOT in the nanocavity is now probed with DF spectroscopy by integrating *e*NPoM samples into a spectro-electrochemical cell with three electrodes (Fig. 1a). DF spectra of *e*NPoMs for shell thicknesses of 2, 4, 9, 14 nm are recorded at potentials ranging from −0.6 to +0.6 V (*vs* Ag/AgCl) in nitrogen-purged 0.1 M NaCl aqueous electrolyte (Fig. 2a). Reversible redox is successfully traced by the shifting of ${\lambda }_{{\rm{c}}}$ on all four samples, however it is striking that a reversal of the shift direction is observed for *e*NPoMs with sub-5 nm shells (Fig. 2b).

The *e*NPoM samples with shells thicker than 5 nm show characteristic NPoM blue shifts upon PEDOT oxidation (−0.6$\to$0.6 V, P^0^$\to$P^2+^), in line with previous reports^16,17^. This is caused by the switch in electronic structure and the resulting absorption change when it transforms into the more conductive state^22^. However, the electrochemical response in DF spectra is markedly different when reducing the shell thickness below 5 nm, as the coupled mode position now red-shifts upon oxidation (either reversing permittivity changes or decreasing gap size *cf* Eq.1). This transition from blue-shift to red-shift occurs between 6-8 nm thicknesses, where the spectral tuning range (Fig. 2c) and intensity switching (Fig. 2d) are both smallest.

During this electrochemical redox process, small anions enter and leave the thin PEDOT layer to balance the polymer backbone charge, which induces expansion and contraction of the PEDOT layer. To examine whether the reversed spectral tuning originates from kinetic factors such as migration and doping of ions, we replace the electrolyte with 0.1 M NaNO_3_, and repeat the experiments for three different scan rates (50 mV s^−1^, 10 mV s^−1^, 5 mV s^−1^) on *e*NPoM samples with 4 and 14 nm gaps (Fig. S3a, b). The planar NO_3_^-^ ions migrate more slowly compared to smaller Cl^-^ ions, while molecular dynamic simulations^23^ predict poorer intercalation within the PEDOT network. Comparing the spectro-electrochemical response for both gap thicknesses shows no change in spectral tuning direction when varying either electrolyte (Fig. 2e) or scan rate (Fig. 2f). This indicates ion intercalation, and their induced changes in gap thickness and permittivity, are not the cause of reversed tuning. For both thicknesses, the oxidative DF tuning ${\Delta \lambda }_{{\rm{c}}}$ changes only slightly with lower scan rates (Fig. S3c). The hysteresis observed (Fig. 2e) is consistent with ion intercalation of PEDOT films for small anions^24,25^, here observed now on the few-nm scale. We note the effect of confinement is to shift the hysteresis to more negative potential by ~−0.4 V. We also observe a slow drift of the plasmon coupled mode over multiple cycles in addition to the strong shifts upon redox cycling (Fig. 2a). This gradual shift is likely attributable to the slow reshaping of the nanoparticle facet, a phenomenon commonly observed under laser illumination^26^.

**Fig. 3:**
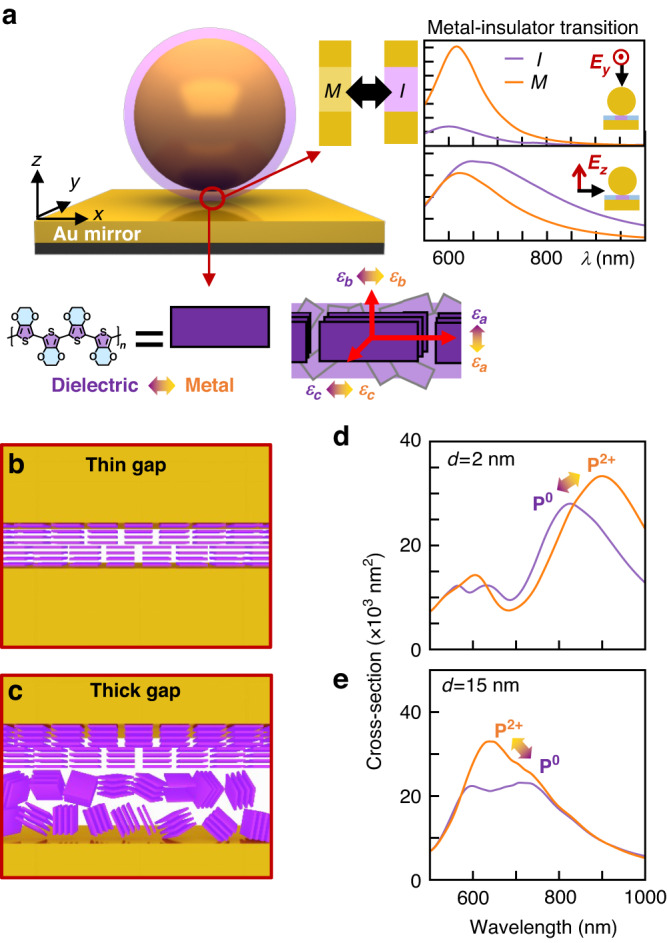
Metal-insulator transition induced optical anisotropy of PEDOT and FDTD simulations. **a** Schematic illustration of *e*NPoM structure, orientated along ${\boldsymbol{x}}{\boldsymbol{,}}{\boldsymbol{y}}{\boldsymbol{,}}{\boldsymbol{z}}$ axes. Right inset: metal-insulator transition in nanogap and simulated scattering spectra using Drude model for different field orientations, ${{\boldsymbol{E}}}_{{\boldsymbol{y}}{\boldsymbol{,}}{\boldsymbol{z}}}$. Below inset: birefringence of PEDOT crystallites, along local ${\boldsymbol{a}}{\boldsymbol{,}}{\boldsymbol{b}}{\boldsymbol{,}}{\boldsymbol{c}}$ axes. ${{\boldsymbol{\varepsilon }}}_{{\boldsymbol{a}}{\boldsymbol{,}}{\boldsymbol{b}}{\boldsymbol{,}}{\boldsymbol{c}}}$: complex permittivity of PEDOT along each axis in dielectric (PEDOT^0^, purple) and metallic (PEDOT^2+^, orange) states. (b,c) Possible PEDOT crystallite orientations close to gold interfaces for **b** ${\boldsymbol{d}}$<5 nm and **c** >5 nm thick coatings. **d**, **e** FDTD simulations of optical scattering cross-section for redox states in thick and thin PEDOT *e*NPoMs with vertical (${{\boldsymbol{E}}}_{{\boldsymbol{z}}}$) field orientation (for model see text)

We first consider the expected tuning effect when the contents of the plasmonic gap change from dielectric to conducting. We used a Drude model for PEDOT in Finite-Difference Time-Domain (FDTD) full-wave simulations (Methods, Fig. 3a, S4) since analytical models discussed above do not account for wavelength-dependent permittivity of the gap materials. For large (15 nm) gaps, in-plane optical fields (${E}_{x,y}$, as excited also by normally-incident light) excite only the single-particle NP plasmon and modulate the scattering cross-section through the metal-insulator transition. By contrast, high-angle light with a vertical optical field component (${E}_{z}$) couples to the gap plasmon confined inside the nanogap for a dielectric spacer. Passing through the metal-insulator transition now shorts out the image charges at either side of the gap, reducing gap capacitance and blue-shifting the plasmon mode (Fig. 3a). This accounts for the thick-film tuning which indeed blue-shifts (Fig. 2b). Even an isotropic gap material exhibiting a metal-insulator transition thus gives an anisotropic response for different optical field orientations in the nanogap.

Besides kinetic factors during PEDOT electrochemical redox, optical anisotropy also arises from the PEDOT microstructure which affects the DF spectra of NPoM devices. PEDOT is rather insoluble in most solvents and typically forms short oligomeric chains during synthesis^27^. Films are known to be paracrystalline^28,29^ with short oligomeric chains (containing 5 to 15 monomeric units^1^) stacked on each other through $\pi$-$\pi$ interactions. These form small lamellar crystallites ranging from 4 to 10 nm^28–30^ embedded in a less-ordered matrix (Fig. 3a), depending on the synthesis^10,31–33^. The optical permittivity thus varies with polarization direction of the optical fields: ${\varepsilon }_{a}$ along the aromatic backbone, ${\varepsilon }_{b}$ perpendicular to the backbone, and ${\varepsilon }_{c}$ along the $\pi$-$\pi$ stacking direction^2,3,34^. This implies that the metal-insulator transition is predominantly for fields along the $a$-axis, as it governs the conductivity. As we show below, our data suggests the microstructures are oriented differently in *e*NPoMs with thin *vs* thick PEDOT shells (Fig. 3b, c). In thin films (<5 nm), the attachment of PEDOT crystallites to gold surfaces predominantly occurs in a face-on orientation. However, as the film thickness increases, PEDOT crystallites located further away from the surface tend to exhibit a more isotropic orientation, which is likely accompanied by a decrease in crystallinity^32^.

FDTD simulations using the full PEDOT anisotropic permittivity (Methods, Fig. S5) from literature^2,3^ allow us to simulate the tuning of DF spectra during redox of PEDOT. For thick gap films (Fig. 3e), a blue-shift is predicted as observed, assuming PEDOT crystallites are disordered and with dominant optical field normal to the Au mirror^11^ (along $z$ axis of NPoM geometry). This is because in this spectral region, the isotropic average permittivity (enhanced by the HOMO-LUMO resonance at 610 nm in the P^0^ state) diminishes when PEDOT is oxidized into the conducting state (Figs. S5, S6). Note that in the core-shell model, both illumination directions predict blue-shifts (Fig. S7). Contrastingly, for thin films (Fig. 3d) a switch in spectral shift directly resolves the non-backbone PEDOT permittivity (${\varepsilon }_{b,c}$) seen by the optical field upon oxidation (Fig. S8). Both the $b$ and $c$ axes of PEDOT exhibit non-metallic dielectric transitions and, while ${\varepsilon }_{b}$ is known to increase upon oxidation, we assume that this is similar for ${\varepsilon }_{c}$. The metal-insulator transition for ${\varepsilon }_{a}$ is thus not seen in small gaps, given the face-on crystallite orientation and vertical $z$ field. Analysis of SERS data below further confirms this face-on orientation of the PEDOT crystallites, with ${\varepsilon }_{c}$ aligned along the optical field ($z$ axis).

### Orientation detection and redox reaction tracking

**Fig. 4:**
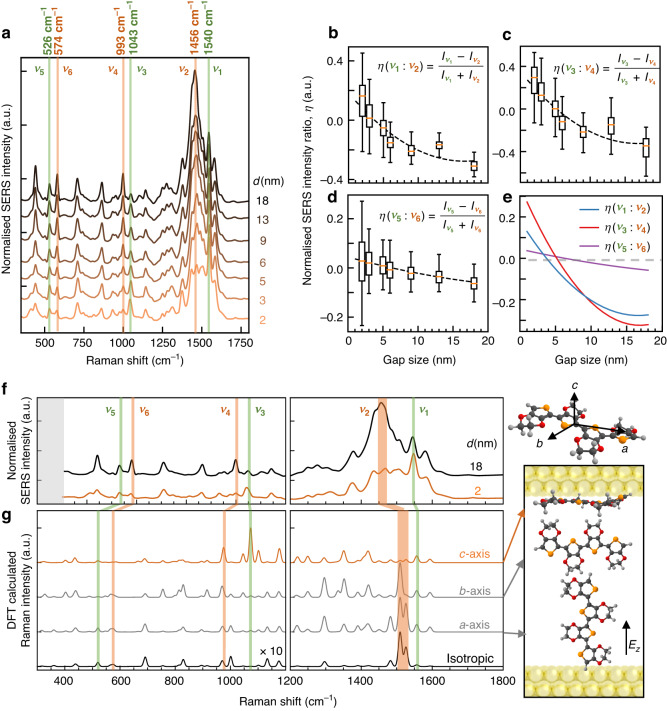
Raman evolution for PEDOT films and dependence on molecular orientation. **a** Average *e*NPoM SERS spectra *vs* shell thickness ${\boldsymbol{d}}$. **b**–**d** Peak intensity ratios ${\boldsymbol{\eta }}$ *vs* shell thickness ${\boldsymbol{d}}$ for **b** 1540:1456 cm^−1^, **c** 1043:993 cm^−1^, and **d** 526:574 cm^−1^ peaks, labelled in **a**. Errors cover range from NPoMs. **e** 2^nd^ order polynomial fits of ${\boldsymbol{\eta }}$ vs PEDOT shell thickness. **f** SERS spectra for NPoMs with ${\boldsymbol{d}}$ = 2, 18 nm PEDOT shells. Inset to right: single PEDOT^2+^ tetramer structure used in DFT for optimized SERS spectra, axes as labelled. **g** DFT simulated Raman spectra of PEDOT^2+^ tetramer separated into components for different optical field polarizations along ${\boldsymbol{a}}{\boldsymbol{,}}{\boldsymbol{b}}{\boldsymbol{,}}{\boldsymbol{c}}$ directions of PEDOT tetramer, as in Fig. 3a. ${{\boldsymbol{E}}}_{{\boldsymbol{z}}}$: Dominant vertical optical field in *e*NPoM nanogap

DF spectra during in-situ CV clearly resolves optical anisotropy in *e*NPoMs with different PEDOT thickness. To better understand the microstructure of PEDOT within such metallic gaps, SERS spectra of *e*NPoMs with 2-18 nm shell thickness are now compared. A 633 nm laser illuminates individual NPoMs for over 200 s per time scans at optical powers ≤ 6 *μ*W to avoid damage (Methods). SERS spectra from different *e*NPoMs of the same PEDOT thickness show strong consistency (Fig. S9). Comparing average SERS spectra for *e*NPoMs of decreasing PEDOT thickness (Fig. 4a) reveals relative peak intensity changes in three regions, now discussed in turn.

The corresponding vibrational modes^35–40^ can be assigned using density functional theory (DFT) frequency calculations (Table S1). A SERS band at 1456 cm^−1^ (${v}_{2}$) associated with the characteristic symmetric C_α_ = C_β_ stretching from PEDOT^2+^ dominates in *e*NPoMs with ${\boldsymbol{d}}$=18 nm, confirming the as-synthesized PEDOT starts in the oxidized state^1^ at $\simeq$30% doping level^17^. This band gradually weakens in samples with thinner PEDOT coating. In contrast, the intensity of the PEDOT^2+^ asymmetric C_α_ = C_β_ stretching band at 1540 cm^−1^ (${v}_{1}$) remains relatively stable irrespective of PEDOT thickness. Defining the relative ratio of lines ${\nu }_{\mathrm{1,2}}$ as $\eta \left({\nu }_{1}:{\nu }_{2}\right)=\left({I}_{1}-{I}_{2}\right)/({I}_{1}+{I}_{2})$ for SERS peak maxima $I$, we find $\eta$(${v}_{1}$:${v}_{2}$) shows a clear sign inversion around ${\boldsymbol{d}}$ = 5 nm (Fig. 4b). A second set of bands at 993 (${v}_{4}$) and 1043 cm^−1^ (${v}_{3}$) show a similar inversion in relative peak intensities $\eta$(${v}_{3}$:${v}_{4}$) from −0.4 to +0.4 (Fig. 4c). The former band is associated with oxyethylene ring deformation, and initially dominates for ${\boldsymbol{d}}$ = 18 nm but weakens in thinner films. By contrast, the SERS band at 1043 cm^−1^ (${v}_{3}$, previously unassigned) strongly increases for thinner films. A third set of bands at 574 (${v}_{6}$) and 526 cm^−1^ (${v}_{5}$) both weaken for smaller$\,{\boldsymbol{d}}$, but at different rates giving a sign change also for $\eta$(${v}_{5}$:${v}_{6}$) (Fig. 4d). Here, the film thickness more significantly influences the 574 cm^−1^ (${v}_{6}$) SERS mode, associated with another oxyethylene ring deformation.

Comparing the peak intensity ratios for all three bands (Fig. 4e) reveals that they invert sign at thickness ~5 nm, consistent with the crystallite re-orientation posited above. Changes in relative SERS peak intensity can be attributed to multiple factors, including changes in PEDOT charge state (and polarizability) and changes in orientation of PEDOT crystallites with respect to the optical field. The strong change in $\eta$(${v}_{3}$:${v}_{4}$) suggests the oxyethylene ring deformation Raman tensor is preferentially oriented along the backbone (*a*-axis). The thinnest 2 nm films show significantly different SERS spectra in the 1300-1700 cm^−1^ range compared to thicker PEDOT (Fig. S10). Their SERS peaks now become unstable and fluctuate over time (due to so called ‘picocavities’), which is characteristic when the extreme-confined light generates optical forces that become sufficient to create Au adatoms which transiently interact with the molecules^41^.

To examine the influence of PEDOT charge state on these different vibrations, electrochemical scans with in-situ SERS measurement are performed on *e*NPoM samples of ${\boldsymbol{d}}$ = 4, 20 nm (Fig. S11). For 20 nm thick PEDOT (Fig. S12a), a SERS band at 1433 cm^−1^ associated with symmetric C_α_ = C_β_ stretching from PEDOT^0^ dominates the spectrum at $V$= −0.6 V. Scanning the potential towards +0.6 V, this band weakens and the PEDOT^2+^ characteristic ${\nu }_{2}$ = 1456 cm^−1^ peak (symmetric C_α_ = C_β_ stretch) emerges (Fig. S12b(i)). The ratio $\tfrac{1}{2}\left[\eta \left(1456:1433\right)+1\right]$ directly tracks the fraction of PEDOT^2+^ as the potential is scanned, indicating the varying oxidation level (Fig. S12c(i)). Similar evolution is seen in the asymmetric C_α_ = C_β_ stretching bands of PEDOT^0^ (1513 cm^−1^) and PEDOT^2+^ (1540 cm^−1^) (Fig. S12b, c(ii)), which again confirms electrochemical oxidation of the PEDOT shell, consistent with literature^17,35,38^. Comparing also the $\eta$(${v}_{1}$:${v}_{2}$), $\eta$(${v}_{3}$:${v}_{4}$) and $\eta$(${v}_{5}$:${v}_{6}$) ratios whose sign tracks crystallite orientation (see above), these remain negative, confirming they are independent of oxidation state (Fig. S13).

By contrast, *e*NPoMs with ${\boldsymbol{d}}$ = 4 nm PEDOT (Fig. S11b) show very different behavior in electrochemical cells. Their SERS exhibits ten-fold lower intensity in the presence of electrolyte, making most bands undetectable. Instead, transient picocavity events become frequent, even at this low laser power ($\sim$10 $\mu$W). Rather than capturing the redox reactions of PEDOT in the gap, these spectral changes likely trace how Au adatoms coordinate with the polymer and induce charge redistribution when electrochemical potential is applied. This offers an interesting regime for future study of conducting polymers at metal interfaces.

Using DFT, the origin of the differences between thick and thin gap *e*NPoM samples can be associated with particular orientations of PEDOT chains in the nanocavity (Fig. 4f, g). As noted above, the optical field in sub-5 nm gap is always oriented perpendicularly to the facets due to the field boundary conditions. The relative orientation of PEDOT molecules relative to this field in the gap thus strongly affects which Raman selection rules deliver PEDOT fingerprints when the shell thickness reduces. Comparing the experimental observations with DFT simulations, *e*NPoMs with a thick PEDOT shell (18 nm) show good agreement with isotropic PEDOT molecules, implying no preferential crystallite orientation. For thin PEDOT shells (2 nm), simulations indicate PEDOT molecules in the gap experience enhanced optical fields along their $c$ axis, which increases intensity of the 1043 cm^−1^ mode associated with oxyethylene ring deformation and deactivates the 1456 cm^−1^ band. Since the optical field is no longer along the $a$-axis which exhibits the metal-insulator transition, the opposite spectral shift direction is explained.

Using SERS to study thin PEDOT thus reinforces the findings obtained from in-situ electrochemical DF spectroscopy. The results consistently indicate that near the Au nanoparticle facets, the polymer orients preferentially with crystallites flat to the Au surface. As the thickness of the film increases, the polymer properties become more isotropic, indicating a greater presence of randomly oriented crystallites and decrease in crystallinity. The critical thickness for this transition in PEDOT morphology, as observed optically using the *e*NPoM geometry in both SERS and DF, is found to be $\sim$5 nm. This suggests that the surface interactions reach many layers into the polymer, and that crystallites are up to 4 layers thick^28^, smaller than previously suggested. We highlight that our study has not yet reached the resolution limit ($d$<1 nm)^42,43^ of the NPoM geometry with the limit for lateral spatial confinement^11^ $\sim \sqrt{{Dd}}/n \sim 4$ nm (for gap refractive index $n \sim$2 and 40 nm NP diameter).

## Conclusion

We demonstrate the use of plasmonic nanogap *e*NPoM architectures to study thin polymer films on metal surfaces. In this architecture, it becomes possible to dynamically study the anisotropic metal-insulator transition of materials inside plasmonic nanogaps. Using 2-18 nm thick PEDOT coatings and systematically studying the redox process in these nanocavities via in-situ spectro-electrochemisty, we find that both DF and SERS data show drastic spectral changes for sub-10 nm polymer thickness. A critical optical transition thickness for Au@PEDOT is identified $\sim$5 nm. Using FDTD and DFT simulations explains both a surprising reversal in DF tuning direction and SERS relative peak intensities. Both DF and SERS data suggest the PEDOT conformation changes from isotropic to face-on orientation when close to the nanoparticle interface. The lateral resolution (depending on gap size^11^) can be <5 nm, comparable to the crystallite scale. This ability to assess the orientation of polymers down to sub-10 nm scales by optical means provides a new methodology to explore material interfaces and facilitate the control and engineering of functionality. It is promising for broad applicability across many other anisotropic materials, such as polymers and two-dimensional materials. This is particularly important in a wide range of fields including surface science, molecular electronics, electrochemistry, catalysis, and organic optoelectronics, and opens up new prospects in nanophotonics.

## Materials and methods

### Au@PEDOT core–shell nanoparticles synthesis

PEDOT-coated Au nanoparticles were synthesized using surfactant-assisted chemical oxidative polymerization. 1.6 mL of 100 nm gold nanoparticles in citrate buffer solution (BBI Solutions) was concentrated and mixed with 0.6 mL of 3,4-ethylenedioxythiophene (EDOT, monomer) aqueous solution and 0.12 mL of 40 mM sodium dodecyl sulfate (SDS) solution. Then 0.6 mL of 2 mM ammonium persulfate (polymerization initiator) solution and 8.25 μL of 20 mM iron chloride (catalyst) solution was added to the mixture. The mixture was incubated at room temperature for 14 h to allow growth of the PEDOT shells, and the final core-shell nanoparticles were washed and stored in 1.6 mL of 4 mM SDS solution at 2-5 °C. The PEDOT shell thickness is controlled by adjusting the concentration (0.5, 1.0, 1.5, 2.0, 2.5, 3.0, 4.0, 6.0, 8.0, and 10.0 mM) of monomer solution added.

### *e*NPoM sample preparation

The *e*NPoM samples were prepared by drop-casting 3 μL of the core-shell nanoparticle solution on a template-stripped Au substrate for 60 s, with 0.5 μL of 1 M NaNO_3_ aqueous solution added to the droplet to assist deposition. The samples were rinsed with distilled water and then blow dried using dry nitrogen gas.

### Spectro-electrochemical cell

All spectro-electrochemical measurements were performed in a custom-designed three-electrode electrochemical cell, where *e*NPoM substrates, a platinum mesh (Alfa Aesar) and Ag/AgCl (3 M KCl, eDAQ ET072, Green Leaf Scientific) were used as working, counter, and reference electrodes respectively. A 25$\times$25$\times$0.2 mm^3^ glass coverslip was adhered to the top of the cell to create a fluid chamber. All the electrochemical measurements were operated and recorded using a CompactStat potentiostat (Ivium Technologies).

### DLS and EM characterization

A zeta potential analyzer (Zetasizer Nano ZS, Malvern) was used to measure the DLS spectra of 0.1 mL Au@PEDOT solution, where physical properties of gold and water were used as input parameters. Standard deviation of the shell thickness is calculated as $\sigma =\frac{1}{2}\sqrt{{\sigma }_{{after}}^{2}+{\sigma }_{{before}}^{2}}$. All SEM images were captured using the FEI Helios NanoLab Dual Beam microscope, while TEM images were captured using the FEI Tecnai F20 transmission electron microscope.

### Optical imaging and spectroscopy

A customized microscope (Olympus BX51) with a halogen white light lamp, a charge-coupled device camera (Infinity 2), spectrometer (Ocean Optics QE65000), and a 100$\times$ objective (NA 0.8 Olympus LMPLFLN) was used to capture optical DF images and spectra. All the DF spectra were integrated for 1 s and normalized using white light scattering of a standard diffuser. SERS spectra were recorded on the same microscope with a 633 nm laser for excitation, a 100$\times$ objective (NA 0.9 Olympus LWD) and an Andor Newton EMCCD camera coupled to a Horiba Triax 320 spectrometer. For non-electrochemical SERS measurements on as-synthesized *e*NPoMs (in air), the laser power was set at 6 $\mu {\rm{W}}$ on 2 nm shell samples, and 3 $\mu {\rm{W}}$ on all other shell thickness NPoMs, for optimal signal-to-noise ratios and minimization of picocavity events. For electrochemical SERS measurements, the laser power was set to 10 $\mu {\rm{W}}$ for all samples. The integration time of all SERS measurements was 1 s if not otherwise specified. For non-electrochemical DF and SERS measurements, automated scans were performed using customized particle-tracking Python code.

### Simulations for electromagnetic and DFT calculations

Finite-difference time-domain (FDTD) calculations were performed using Lumerical Solution to simulate the electromagnetic response of the *e*NPoM system. For the gold nanoparticle and mirror, we utilized the gold permittivity measured by Johnson and Christy. For Drude-dielectric switching of PEDOT, a dielectric material with $n=1$ and $k=0$ and a Drude metal is used to simulate the reduced and oxidized states of the PEDOT backbone (*a*-axis) respectively. Complex permittivity of the Drude metal is defined in the SI. For the anisotropic PEDOT model, the permittivity of anisotropic PEDOT is adapted from literature^2,3^ where the in-plane permittivity is $0.5\left({\varepsilon }_{a}+{\varepsilon }_{c}\right),$ and out-of plane permittivity is ${\varepsilon }_{b}$ as reported for an edge-on PEDOT film. The isotropic permittivity is calculated as $0.33\left({\varepsilon }_{a}+{\varepsilon }_{b}+{\varepsilon }_{c}\right)$, for simplicity. However, it should be noted that more comprehensive methods for calculating composites of metallic and dielectric materials may be required^44^.

DFT calculations were performed on either the isolated molecule, or the molecule bound to a single gold atom to approximate binding to a gold surface. Gas-phase geometry optimizations and frequency calculations were performed using the B3LYP^45,46^ hybrid generalized gradient approximation exchange-correlation functional, augmented with Grimme’s D3 dispersion correction with Becke-Johnson damping (GD3BJ)^47^. Unrestricted self-consistent field calculations were performed in all cases, to ensure electronic stability of the wavefunction. The Def2SVP^48^ basis set was employed for all atoms and included a pseudopotential for Au. All DFT calculations were implemented with an ultrafine integration grid, without symmetry restrictions, using Gaussian 09 Rev. E.^49^ Directional components of DFT-calculated Raman spectra were extracted from the polarizability tensor as described by Le Ru & Etchegoin^50^ and Grys et al.^51^. All the calculated SERS spectra were frequency scaled by a factor of 0.9671^52^.

### Supplementary information

Supplementary Information

## Data Availability

The data that support the findings of this study are available at 10.17863/CAM.104448.
