# Supplementary material for: Metal to insulator transition for conducting polymers in plasmonic nanogaps
Source: Light Sci Appl. 2024 Jan 1;13:3. doi: 10.1038/s41377-023-01344-7 (PMC10757999; doi:10.1038/s41377-023-01344-7)
Supplement: Supplementary file 1 — Supplementary Information [file 41377_2023_1344_MOESM1_ESM.pdf]

# Supplementary Information

# Metal to insulator transition for conducting polymers in plasmonic nanogaps

Yuling Xiong[1], Rohit Chikkaraddy[1,2], Charlie Readman[1], Shu Hu[1], Kunli Xiong[1], Jialong Peng[1,3], Qianqi Lin[1,4], and Jeremy J Baumberg[1*]

[1] Nanophotonics Centre, Cavendish Laboratory, University of Cambridge, Cambridge, CB3 0HE, UK

[2] School of Physics & Astronomy, University of Birmingham, Edgbaston, Birmingham, B15 2TT, UK

[3] College of Advanced Interdisciplinary Studies and Hunan Provincial Key Laboratory of Novel Nano-Optoelectronic Information Materials and Devices, National University of Defense Technology, Changsha 410073, China

[4] Hybrid Materials for Opto-Electronics Group, Department of Molecules and Materials, MESA+ Institute for Nanotechnology, Molecules Center and Center for Brain-Inspired Nano Systems, Faculty of Science and Technology, University of Twente, 7500 AE Enschede, Netherlands

* emails: jjb12@cam.ac.uk

**Contents of Supplementary Information:**

Note S1: Prediction of coupled mode wavelength: Circuit Model vs QNM model

Fig. S1: PEDOT thickness measured by DLS
Fig. S2: Field distribution for typical resonance wavelength in eNPoMs
Fig. S3: Optical switching of Au@PEDOT *e*NPoM
Fig. S4: Drude model of used for metallic state
Fig. S5: Anisotropy in PEDOT permittivity at different charge states
Fig. S6: Calculated DF scattering spectra of NPoM with 15 nm thick isotropic PEDOT disc
Fig. S7: Calculated DF scattering spectra of *e*NPoM with 15 nm thick isotropic PEDOT shell
Fig. S8: Calculated DF scattering spectra of NPoM with anisotropic PEDOT disc
Fig. S9: SERS spectra for *e*NPoMs at different shell thickness
Fig. S10: Comparison of time-scan SERS spectra for 2 nm and 13 nm gap PEDOT shell
Fig. S11: In situ cyclic-voltammetry SERS time-scan spectra of *e*NPoMs
Fig. S12: Raman evolution of *e*NPoM with 20 nm shell during redox
Fig. S13: Analysis of SERS dynamics of peak of interets ($v_1$-$v_6$) during redox

Table S1: Assignments of characteristic Raman bands for PEDOT

## Note S1 | Prediction of coupled mode wavelength: Circuit Model vs QNM model

The circuit model (main text Eqn.1) can predict the general tuning direction of the coupled mode wavelength in response to changes in gap material permittivity and gap size. However, it does not account for nanoparticle facet size and shape, which can also influence the optical coupled modes in NPoMs. A more precise (and recent) model exploits the Quasi-Normal Mode (QNM) decomposition of NPoMs (see ref[1], and https://www.np.phy.cam.ac.uk/npom-calculator which is an online NPoM mode calculator). This method considers facet shape (circular, triangular, square) and facet fraction ($f = w/D$, where $w$ is facet diameter, and $D$ is NP diameter). The circuit model, the QNM decomposition method (circular facet, $f$= 0.35), and experimental data from **Fig.1e** are compared in **Fig. S2b** below. These simplistic models miss another point: without external potential, PEDOT is partially oxidised, giving a strong resonance between 550-750 nm in the real part of its permittivity (**Fig.S5**). These features explain why such models cannot exactly predict the experimental data.

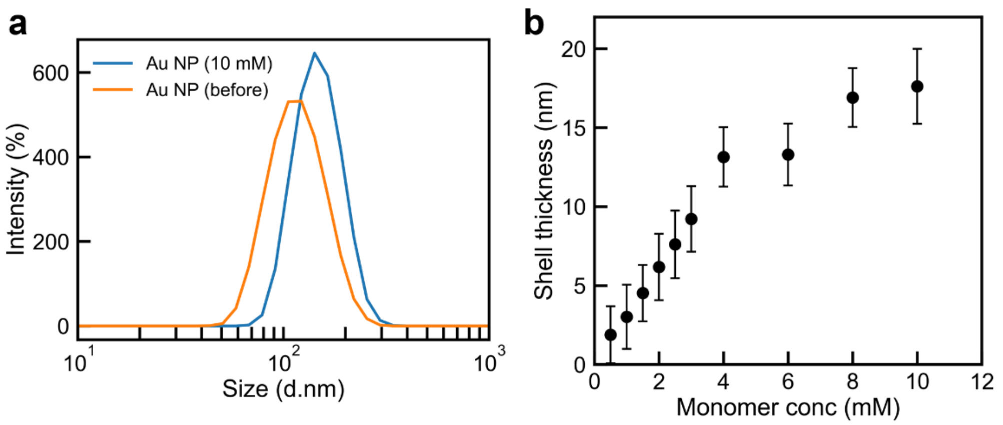


**Fig. S1 | PEDOT thickness measured by DLS.** (a) Typical nanoparticle size distribution measured using DLS of (citrate capped) gold nanoparticle before and after polymer growth with 10 mM monomer. (b) Calculated PEDOT shell thickness as a function of monomer concentration. Error bar: Standard deviation of polymer shell.

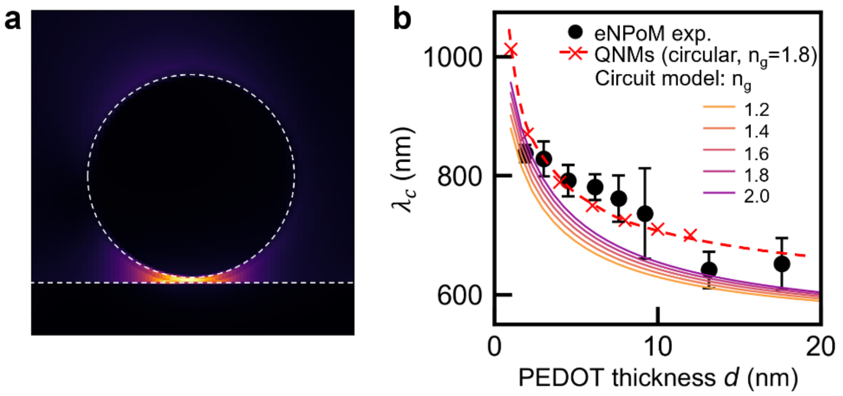


**Fig. S2 |** (a) Field distribution for typical coupled resonance wavelength in *e*NPoMs, 80 nm diameter, 1.5 nm gap size. (b) NPoM coupled mode plasmon $\boldsymbol{\lambda_c}$ vs PEDOT shell thickness $\boldsymbol{d}$ (in air) compared with predictions from the circuit model and QNM model (circular facet, f=0.35). Error bar shows width of $\boldsymbol{\lambda_c}$ histogram.

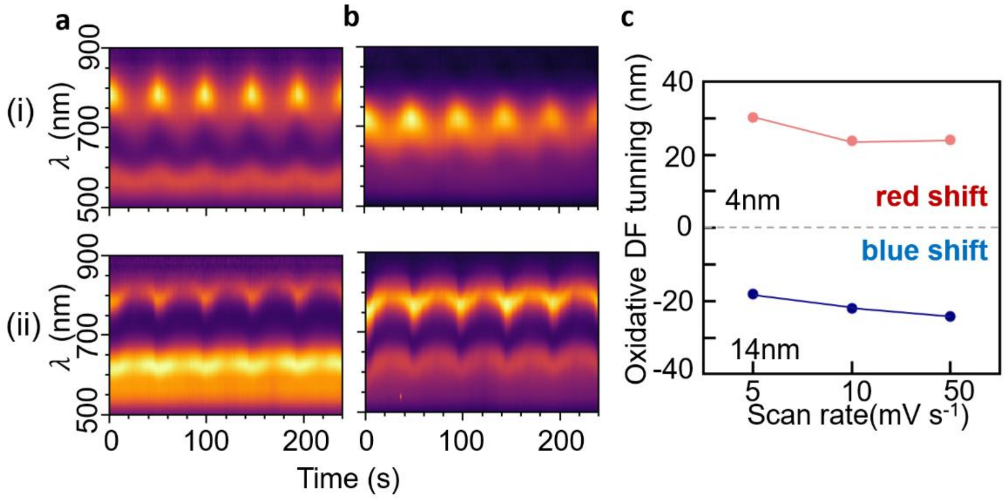


**Fig. S3 | Optical switching of Au@PEDOT *e*NPoM. (i)** Au@PEDOT with 14 nm shell. **(ii)** Au@PEDOT with 4 nm shell. Time-series DF scattering spectra from single *e*NPoMs over 5 cycles in **(a)** 0.1 M NaCl (aq) and **(b)** in 0.1 M $NaNO_3$ (aq). Scan range: -0.6 V ↔ +0.6 V, scan rate: 50 mV $s^{-1}$. **(c)** Oxidative DF tuning *vs* scan rate for Au@PEDOT with 14 nm and 4 nm shells in 0.1 M $NaNO_3$ (aq). Scan range: -0.6 V ↔ +0.6 V.

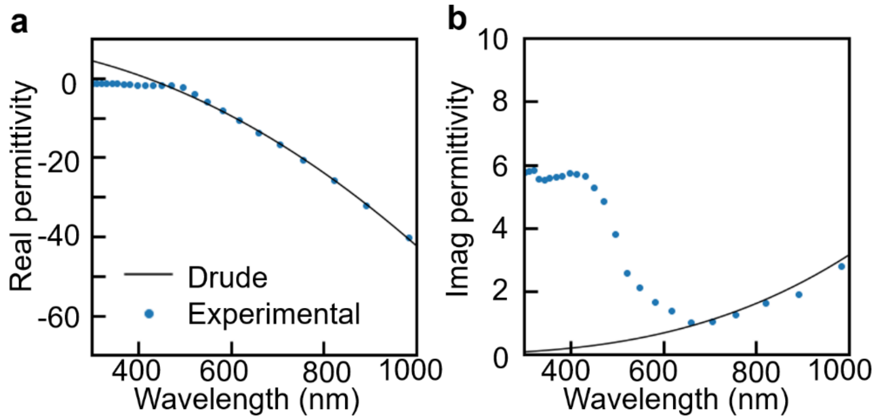


**Fig. S4 | Drude model used for metallic state. (a)** Real and **(b)** imaginary parts of the permittivity used for the metallic phase of the gap in simulation. Points show permittivity of gold measured from Johnson and Christy[2] (copyright Americal Physical Society). Parameters of Drude fitting are adapted from Ref [3].

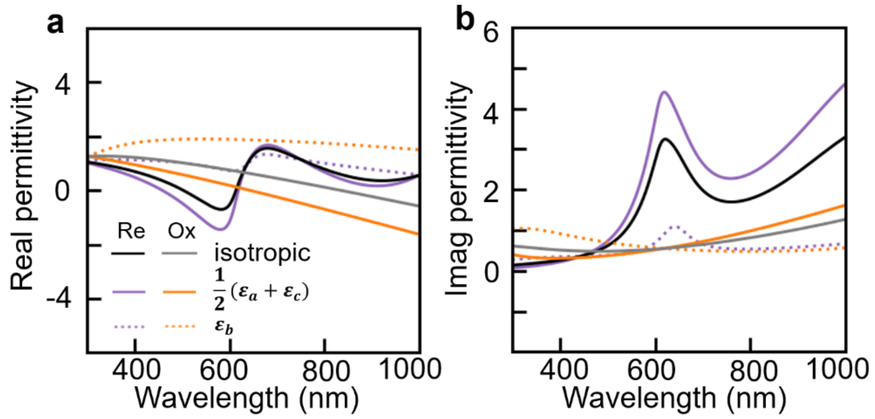


**Fig. S5 | Anisotropy in PEDOT permittivity at different charge state. (a)** Real and **(b)** imaginary parts of PEDOT permittivity along different axes measured using ellipsometry and the calculated isotropic permittivity, $\frac{1}{3}(\varepsilon_a + \varepsilon_b + \varepsilon_c)$. Anisotropic permittivity data are adapted from Ref [4,5] (used with permission). Labels 'ox' and 're' in legend represent PEDOT in $P^{2+}$ and $P^0$ states.

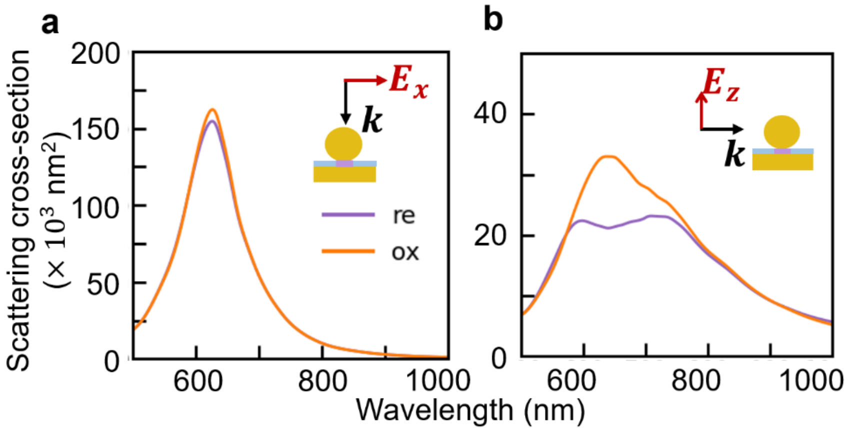


**Fig. S6 |** Calculated DF scattering spectra of NPoM geometry with 15 nm thick PEDOT disc (radius: 15 nm) in the gap in reduced (purple, $P^0$) and oxidised (orange, $P^{2+}$) states. Simulations use two different illumination directions: **(a)** normal and **(b)** high angle illumination. Arrows show $E, k$ directions.

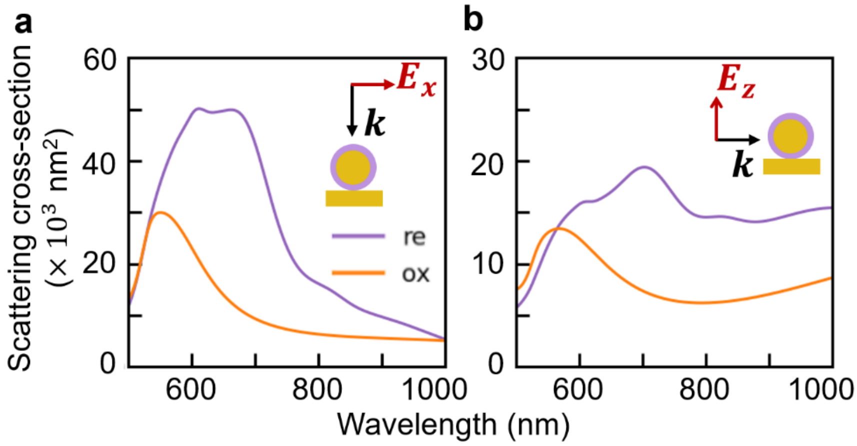


**Fig. S7 |** Calculated DF scattering spectra of *e*NPoM geometry with 15 nm thick isotropic PEDOT shell in reduced (purple, $P^0$) and oxidised (orange, $P^{2+}$) states. Simulations use two different illumination directions: **(a)** normal and **(b)** high angle illumination. Arrows show $E, k$ directions.

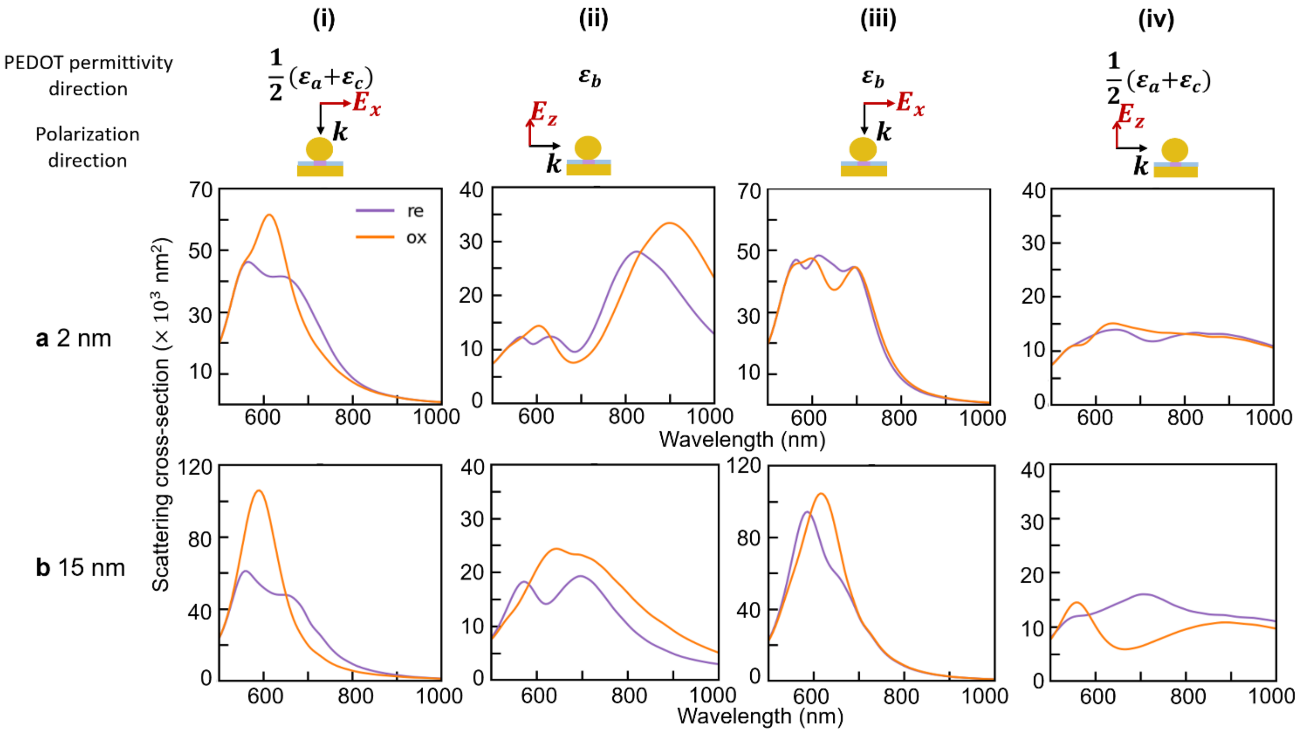


**Fig. S8 |** Calculated DF scattering spectra of NPoM with **(a)** 2 nm and **(b)** 15 nm thick anisotropic PEDOT disc (radius: 15 nm) in the gap for reduced (purple, $P^0$) and oxidised (orange, $P^{2+}$) states. **(i-iv)** show different combinations of two different illumination conditions and PEDOT permittivity seen by the optical field.

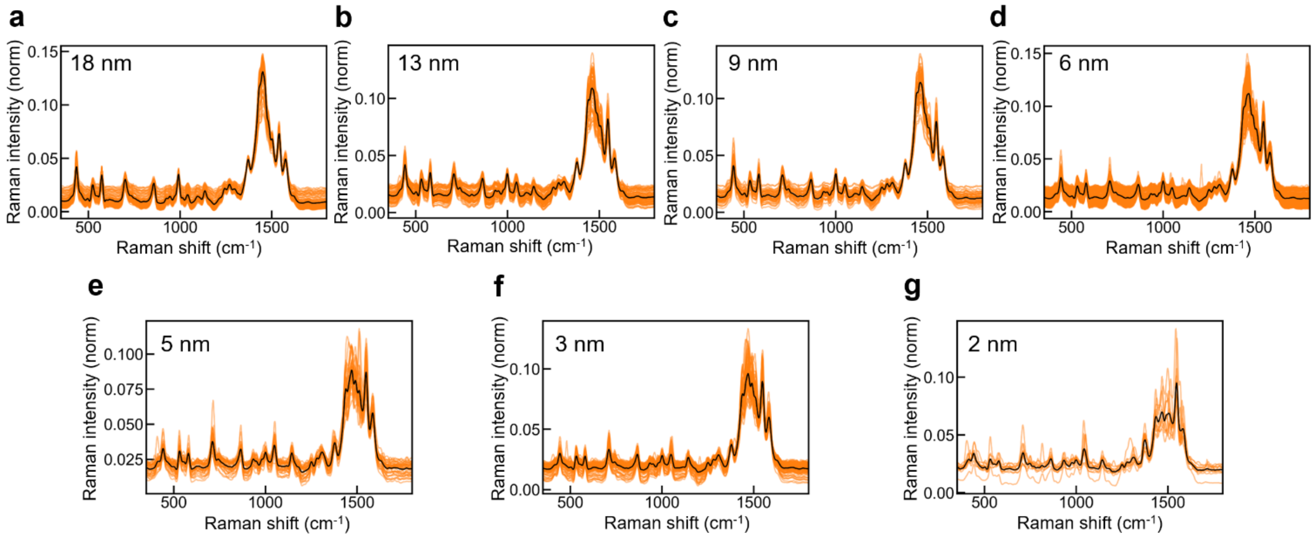


**Fig. S9 |** SERS spectra for *e*NPoMs of different shell thickness. Black line indicates the average of (a) 78, (b) 72, (c) 49, (d) 171, (e) 77, (f) 35 and (g) 12 *e*NPoMs.

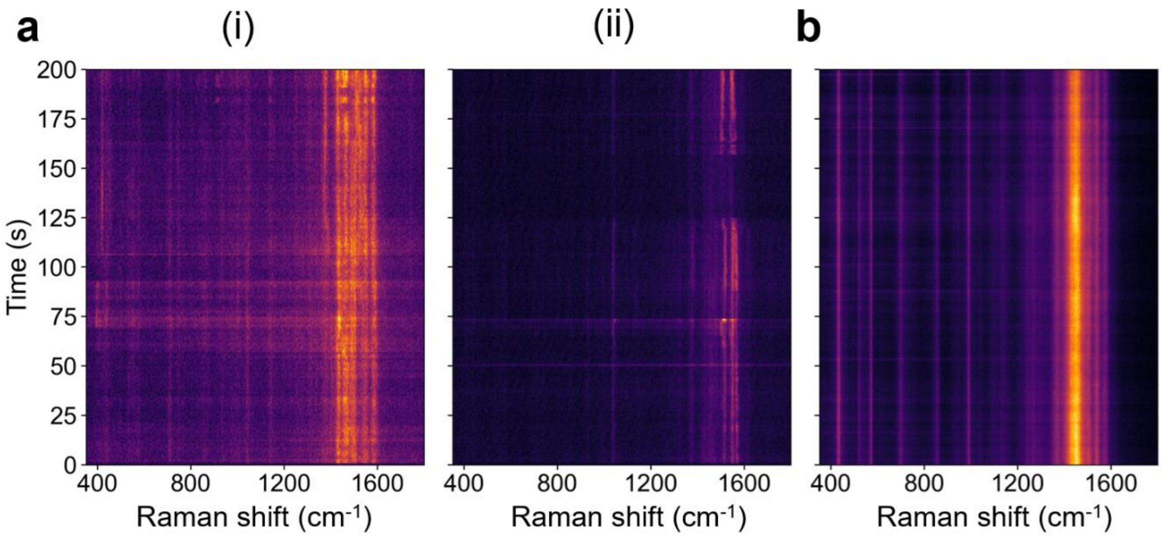


**Fig. S10 | (a)** Example of unstable picocavity SERS spectra collected from two different *e*NPoMs with 2 nm gap. Laser power 6 μW. Integration time: 1 s. **(b)** Example of a stable nanocavity SERS spectra, collected from an *e*NPoM with 13 nm gap. Laser power: 3 μW. Integration time: 1 s.

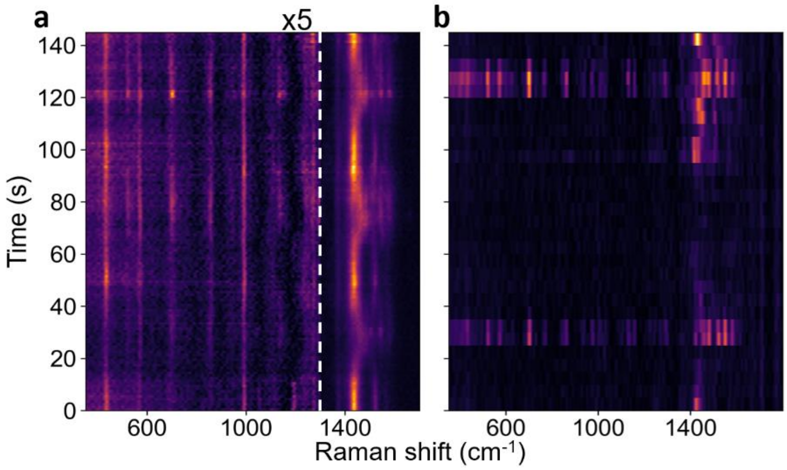


**Fig. S11 |** Time scan of in situ cyclic-voltammetry SERS spectra for *e*NPoMs with **(a)** 20 nm and **(b)** 4 nm thick PEDOT shell for three cycles. Scan range: -0.6 V ↔ +0.6 V, scan rate: 50 mV $s^{-1}$. Integration time: 1 s for 20 nm sample, 5 s for 4 nm sample.

**Table S1 | Assignments of characteristic Raman bands for PEDOT**

| Oxidation state | Wavenumbers ($cm^{-1}$) | Vibrational modes | Ref |
|---|---|---|---|
| 0 → 2+ | 1433 →1456 ($v_2$) | $v(C_\alpha=C_\beta)$ symmetric | 6–8 |
| 0 → 2+ | 1513 → 1540 ($v_1$) | $v(C_\alpha=C_\beta)$ asymmetric | 6–8 |
| 0, 1+, 2+ | $v_3$: 1043, 1031 (in electrolyte) | oxyethylene ring deformation associated with face-on orientation | - |
| 0, 1+, 2+ | $v_4$: 993, 989 (in electrolyte) | oxyethylene ring deformation associated with isotropic orientation | 6–8 |
| 0, 1+, 2+ | $v_6$: 574, 571 (in electrolyte) | oxyethylene ring deformation | 7,8 |
| 0, 1+, 2+ | $v_5$: 526, 524 (in electrolyte) | oxyethylene ring deformation | - |

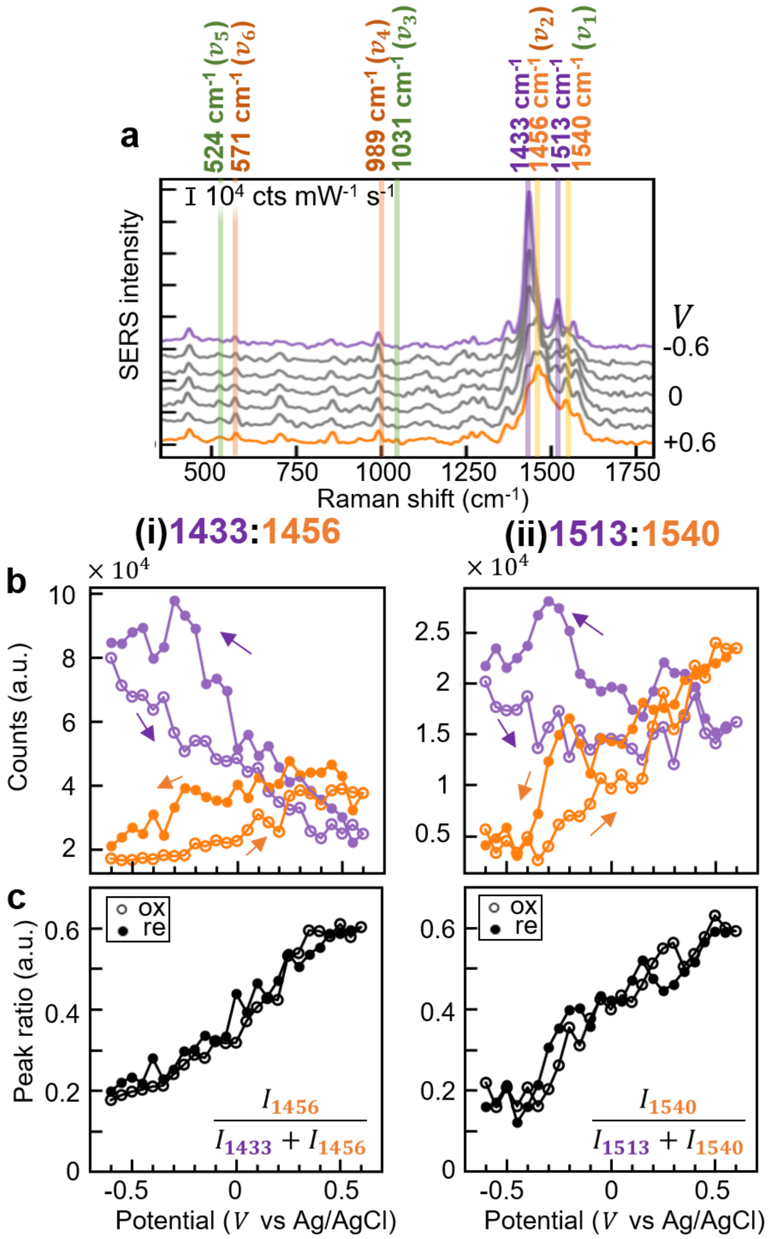


**Fig. S12 | Raman evolution of *e*NPoM with 20 nm shell during redox. (a)** SERS spectra *vs* voltage. **(b)** Extracted peak intensity *vs* applied potential of **(i)** symmetric $\nu(C_\alpha{=}C_\beta)$ for $PEDOT^0$ at 1433 $cm^{-1}$ and $PEDOT^{2+}$ at 1456 $cm^{-1}$, and **(ii)** asymmetric $\nu(C_\alpha{=}C_\beta)$ for $PEDOT^0$ at 1513 $cm^{-1}$ and $PEDOT^{2+}$ at 1540 $cm^{-1}$. **(c)** Fractional ratio of the two components, defined as $I_{ox}/(I_{re} + I_{ox})$ at each V. Labels 'ox' and 're' in legend represent PEDOT oxidation (0.6→-0.6 V, $P^{2+}$→$P^0$) and reduction (-0.6→0.6 V, $P^0$→$P^{2+}$) processes.

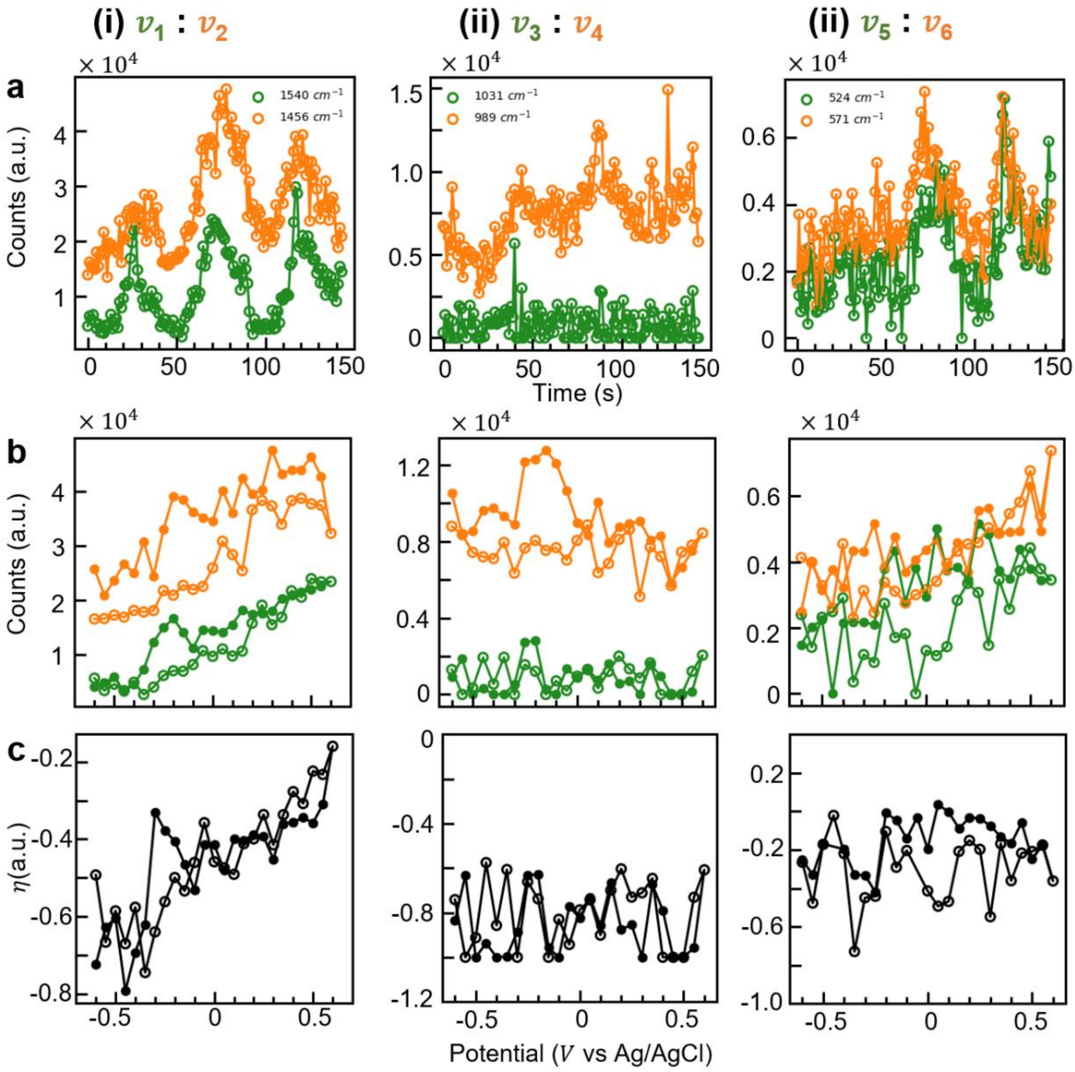


**Fig. S13 | Analysis of SERS dynamics of peak of interets ($v_1$-$v_6$) during redox. (a)** Extracted peak intensity *vs* time and **(b)** *vs* applied potential of **(i)** $v_1$ and $v_2$, **(ii)** $v_3$ and $v_4$ and **(iii)** $v_5$ and $v_6$. **(c)** Peak intensity ratios $\eta$ *vs* applied potential.

## References


1. Elliott, E. *et al.* Fingerprinting the Hidden Facets of Plasmonic Nanocavities. *ACS Photonics* **9**, 2643–2651 (2022).
2. Johnson, P. B. & Christy, R. W. Optical Constants of the Noble Metals. *Physical Review B* **6**, 4370 (1972).
3. Vial, A. *et al*. Improved analytical fit of gold dispersion: Application to the modeling of extinction spectra with a finite-difference time-domain method. *Physical Review B* **71**, 085416 (2005).
4. Chen, S. *et al.* Conductive polymer nanoantennas for dynamic organic plasmonics. *Nature Nanotechnology* **15**, 35–40 (2020).
5. Karki, A. *et al.* Electrical Tuning of Plasmonic Conducting Polymer Nanoantennas. *Advanced Materials* **34**, 2107172 (2022).
6. Garreau, S. *et al*. Optical study and vibrational analysis of the poly(3,4-ethylenedioxythiophene) (PEDT). *Synthetic Metals* **101**, 312–313 (1999).
7. Garreau, S. *et al*. In situ spectroelectrochemical Raman studies of poly(3,4-ethylenedioxythiophene) (PEDT). *Macromolecules* **32**, 6807–6812 (1999).

8. Chiu, W. W. *et al*. Studies of dopant effects in poly(3,4-ethylenedioxythiophene) using Raman spectroscopy. *Journal of Raman Spectroscopy* **37**, 1354–1361 (2006).
